# Immunogenicity and Viral Shedding of Russian-Backbone, Seasonal, Trivalent, Live, Attenuated Influenza Vaccine in a Phase II, Randomized, Placebo-Controlled Trial Among Preschool-Aged Children in Urban Bangladesh

**DOI:** 10.1093/cid/ciy1003

**Published:** 2018-11-27

**Authors:** Kristen D C Lewis, Justin R Ortiz, Mohammed Z Rahman, Min Z Levine, Larisa Rudenko, Peter F Wright, Jacqueline M Katz, Len Dally, Mustafizur Rahman, Irina Isakova-Sivak, Natalia A Ilyushina, Victoria Matyushenko, Alicia M Fry, Stephen E Lindstrom, Joseph S Bresee, W Abdullah Brooks, Kathleen M Neuzil

**Affiliations:** 1 Pharmaceutical Product Development, San Diego, California; 2 Center for Vaccine Development and Global Health, University of Maryland School of Medicine, Baltimore; 3 Infectious Diseases Division, International Centre for Diarrheal Disease Research, Bangladesh, Dhaka; 4 Influenza Division, Centers for Disease Control and Prevention, Atlanta, Georgia; 5 Department of Virology, Institute of Experimental Medicine, St Petersburg, Russia; 6 Dartmouth College, Hanover, New Hampshire; 7 The Emmes Corporation, Rockville; 8 Center for Drug Evaluation and Research, Food and Drug Administration, Silver Spring; 9 International Health, Johns Hopkins University, Baltimore, Maryland

**Keywords:** clinical trials, influenza vaccine, children, Bangladesh

## Abstract

**Background:**

We evaluated a Russian-backbone, live, attenuated influenza vaccine (LAIV) for immunogenicity and viral shedding in a randomized, placebo-controlled trial among Bangladeshi children.

**Methods:**

Healthy children received a single, intranasal dose of LAIV containing the 2011–2012 recommended formulation or placebo. Nasopharyngeal wash (NPW) specimens were collected on days 0, 2, 4, and 7. Reverse transcription polymerase chain reactions and sequencing identified the influenza virus (vaccine or wild-type). On days 0 and 21, blood specimens were collected to assess immunogenicity using hemagglutination inhibition, microneutralization, and immunoglobulin A (IgA) and G enzyme-linked immunosorbent assays (ELISAs); NPW specimens were also collected to assess mucosal immunogenicity using kinetic IgA ELISA.

**Results:**

We enrolled 300 children aged 24 through 59 months in the immunogenicity and viral shedding analyses. Among children receiving LAIV, 45% and 67% shed A/H3N2 and B vaccine strains, respectively. No child shed A/H1N1 vaccine strain. There were significantly higher day 21 geometric mean titers (GMTs) for the LAIV, as compared to the placebo groups, in all immunoassays for A/H3N2 and B (log_10_ titer *P* < .0001; GMT Ratio >2.0). Among immunoassays for A/H1N1, only the mucosal IgA GMT was significantly higher than placebo at day 21 (log_10_ titer *P* = .0465).

**Conclusions:**

Children vaccinated with LAIV had serum and mucosal antibody responses to A/H3N2 and B, but only a mucosal IgA response to A/H1N1. Many children shed A/H3N2 and B vaccine strains, but none shed A/H1N1. More research is needed to determine the reason for decreased LAIV A/H1N1 immunogenicity and virus shedding.

**Clinical Trials Registration:**

NCT01625689.

## Teaser

In this Phase II trial of live, attenuated influenza vaccine in Bangladeshi children, we detected post-vaccination antibody responses to A/H3N2 and B viruses and viral shedding. However, we detected a reduced post-vaccination antibody response to A/H1N1 and no viral shedding.

**(See the Major Article by Brickley et al on pages 786–94, and the Editorial Commentary by Belshe on pages 795–6.)**

Developing influenza vaccines that prevent severe illnesses in children and that are programmatically suitable for use in low- and middle-income countries (LMICs) is a global priority [1, 2]. Live, attenuated influenza vaccines (LAIVs) can potentially address this need. The manufacturing and programmatic attributes of LAIVs also make them desirable products for pandemic responses, with a total global production capacity of 500 million doses of pandemic LAIVs [3]. Head-to-head, randomized clinical trials with inactivated influenza vaccines have demonstrated superior efficacy of LAIVs in young children against seasonal influenza [4, 5], although more recent observational studies have not shown this advantage [6–8]. Given these benefits, the World Health Organization (WHO) created a program to transfer the manufacturing capacity of Russian-backbone LAIV to developing country manufacturers to improve the access of LMICs to seasonal and pandemic influenza vaccines [9, 10].

There are currently 2 different LAIV technologies: (1) Ann Arbor–backbone (produced by AstraZeneca, United Kingdom), sold as FluMist and Fluenz; and (2) Russian-backbone (produced by NPO Microgen, Irkutsk, Russia, and Serum Institute of India), sold as Ultravac and Nasovac-S. Recently, the performance of both LAIV technologies has been variable across studies and countries [7, 11–13]. Some observational studies and laboratory analyses implicate the failure of the FluMist/Fluenz influenza A/H1N1 component [6, 14].

In 2012, we conducted a Phase II, placebo-controlled, randomized clinical trial (RCT) of the Russian-backbone LAIV in children in Bangladesh [15]. This publication reports on the secondary immunogenicity and vaccine-virus shedding objectives.

## MATERIALS AND METHODS

### Study Design

We conducted a Phase II, randomized, double-blind, parallel-group, placebo-controlled RCT of LAIV among children in urban Dhaka, Bangladesh. This publication reports on these secondary objectives: (1) to determine the post-vaccination anti-influenza immunologic responses among children receiving LAIV and placebo; and (2) to determine the post-vaccination shedding of the vaccine viruses among children receiving LAIV and placebo.

### Study Site and Participants

Children 24 through 59 months of age residing in the Kamalapur field site of the International Centre for Diarrheal Disease Research, Bangladesh (icddr,b), participated in this study. Inclusion and exclusion criteria and other aspects of the trial design have been described previously [15]. Generally, healthy children who were not participating in any other intervention studies and who did not have any contraindications to receipt of LAIV were eligible. All participants were influenza vaccine–naive.

### Vaccine

The Serum Institute of India, Ltd. (SIIL) LAIV (Lot 166E2001), containing the 2011–2012 WHO-recommended Northern Hemisphere vaccine strains (A/California/7/2009 [H1N1] pdm09-like virus; A/Victoria/361/2011 [H3N2]-like virus; and B/Wisconsin/1/2010-like virus) and placebo (Lot E9001PCB), were donated by the manufacturer and stored at 2–8^○^C until the time of administration. The lyophilized study vaccine was reconstituted using sterile water diluent at the time of vaccination and 0.25 mL was administered in each nostril (0.5 mL total) using a single-use sprayer. Influenza A/H1N1 and A/H3N2 LAIV concentrations were not less than 10^7.0^ egg infectious dose 50% (EID_50_)/dose and the influenza B component was not less than 10^6.5^ EID_50_/dose. The SIIL LAIV product is licensed for use as a single dose [16].

### Randomization and Blinding

Randomization and blinding procedures have been described previously [15]. On the day of enrollment, following confirmation of eligibility and obtaining parental/guardian consent, participants were randomized in a 1:1 ratio to receive a single dose of LAIV or placebo.

### Clinical Specimens

We collected blood to assess serum immunogenicity and conducted nasopharyngeal washes (NPW) to assess mucosal immunogenicity and post-vaccination viral shedding. Enrolled participants had a blood sample and NPW specimen collected immediately prior to vaccination. Following vaccination, research assistants visited participant homes daily for the first 7 days post-vaccination to monitor safety outcomes. Participants had NPW specimens collected by study physicians on days 2, 4, and 7 to assess vaccine virus shedding. We assumed that the presence of LAIV post-vaccination indicated the vaccine virus infectivity. Participants returned to the study clinic 21 days following vaccination to have post-vaccination blood and NPW specimens collected.

NPW specimens collected on days 0, 2, 4, and 7 post-vaccination were tested for influenza virus (A/H1N1, A/H3N2, and B) by the icddr,b laboratory. Specimen RNA samples were tested using Centers for Disease Control and Prevention (CDC) real-time reverse transcription polymerase chain reaction assays for influenza detection and characterization (procedures available upon request at www.cdc.gov/flu/clsis/). NPW aliquots from days 0 and 21 were shipped to the Geisel School of Medicine at Dartmouth College (Hanover, NH) for mucosal antibody determinations and aliquots from days 0, 2, 4 and 7 were shipped to the Institute of Experimental Medicine (IEM; St. Petersburg, Russia) for further characterization of influenza viruses. Serum aliquots were shipped to the US CDC (Atlanta, GA) for serum antibody determinations. Endpoints in this RCT were based on recommendations by a WHO Consultation on Immunological Endpoints for LAIV Clinical Trials in 2009 [17].

### Vaccine Virus Shedding Procedures

At IEM, day 0, 2, 4, and 7 NPW specimens that had tested positive by reverse real-time polymerase chain reaction for influenza at the icddr,b laboratory were subsequently tested for LAIV virus strains. Wild-type and vaccine-type influenza virus genes were differentiated using Sanger sequencing, as described previously [18]. We refer to LAIV “detections” as laboratory evidence of vaccine strains in clinical specimens, as determined by icddr,b and IEM analyses. Vaccine virus detection was assumed to be due to replication of LAIV in the oropharynx.

### Mucosal Immunogenicity Procedures

At Dartmouth College, all NPW specimens from days 0 and 21 were tested for vaccine immune responses. LAIV strain-specific immunoglobulin A (IgA) enzyme-linked immunosorbent assays (ELISAs) were used to measure vaccine immunogenicity against all 3 vaccine viruses. NPW specimens were tested by kinetic ELISA, using LAIV antigens, to measure strain-specific IgA, expressed as a fraction of the total specimen IgA, as described previously [19].

### Serum Immunogenicity Procedures

At the CDC, all serum specimens from days 0 and 21 were tested for vaccine immune responses. Serum antibodies were measured by hemagglutination inhibition assay (HAI), microneutralization (MN), and serum hemagglutinin immunoglobulin G (IgG) and IgA ELISAs against all 3 vaccine viruses (using wild type A/H1N1pdm09, A/H3N2, and B viruses for HAI and MN and recombinant hemagglutinin (HA) proteins for IgG and IgA ELISAs), according to methods described previously [20, 21].

### Statistical Analyses

The sample size of 300 participants was driven by the safety objectives. Statistical analyses were performed using SAS V.9.3 (Cary, NC). For immunogenicity, the per-protocol analysis population included participants who received the single dose of the study vaccine and had pre- and post-vaccination blood or NPW specimens collected within the protocol-defined periods.

The anti-influenza immunologic responses were measured according to immunoassay (serum HAI, microneutralization, IgA, IgG, or mucosal IgA) and categorized by vaccine virus strain and study arm. Immune responses were parameterized according to (1) geometric mean titer (GMT) and (2) percentages and exact 95% confidence intervals (95% CI) of subjects with ≥4-fold rises from baseline. HAI measures were categorized by baseline serostatus, defined as titers <10 or ≥10. Differences in GMTs at day 21 post-vaccination between study groups were assessed using a Student *t* test of the log magnitude of the immunogenicity response; unadjusted *P* values are presented. Vaccine virus detections were parameterized according to the number and percentage of participants with detectable, vaccine-type virus shedding, both overall and according to post-vaccination day (ie, days 2, 4, 7), by vaccine allocation and virus strain. Agreement between immunoassays and virus shedding was assessed using correlation matrices and a Cohen’s Kappa statistic. We defined the strength of correlation by the absolute value of the Kappa coefficient: poor (0.00–0.20), fair (0.21–0.40), moderate (0.41–0.60), good (0.61–0.80), and very good (>0.80).

This study was approved by the icddr,b (Dhaka, Bangladesh), the Western Institutional Review Boards (Olympia, WA), the Committee for the Protection of Human Subjects at Dartmouth College (Hanover, NH), and CDC Human Subject Research Determination Review. The trial followed International Conference on Harmonization Good Clinical Practice guidelines and was registered with ClinicalTrials.gov (NCT01625689).

## RESULTS

### Participant Flow

**Figure 1.**
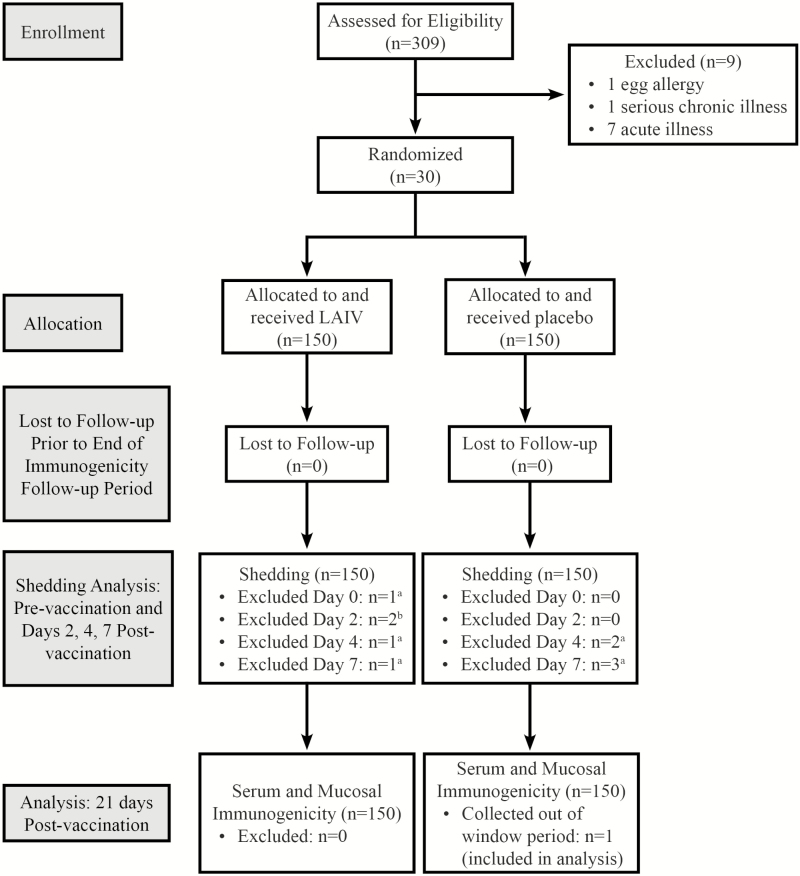
Participant flow. Abbreviation: LAIV, live, attenuated influenza vaccine. ^a^1 specimen not collected and 1 invalid result. ^b^Exclusions due to invalid results.

We enrolled 309 children in the trial over 4 weeks from June to July 2012. There were 300 children eligible for participation, who were randomized to receive either LAIV (n = 150) or placebo (n = 150; Figure 1). All participants remained in the study through the 21-day follow-up immunogenicity outcome period. For the post-vaccination shedding analysis, we excluded results of 10 participants from 1 or more of the pre- or post-vaccination shedding assessments. There was 1 participant in the LAIV group who did not have a specimen collected on day 2 post-vaccination and there were 9 participants who had invalid results on days 0, 2, 4, or 7. There was 1 participant in the placebo group who had a 21-day post-vaccination specimen collected outside the window period, 33 days following vaccination, which was included in the analysis.

### Baseline Characteristics

Baseline characteristics were reported previously [15]. All participants were Bangladeshi and considered generally healthy. There were no differences in the proportion of participants in the LAIV and placebo groups by age, sex, stunting, or use of concomitant medications.

### Immunogenicity Results

**Figure 2.**
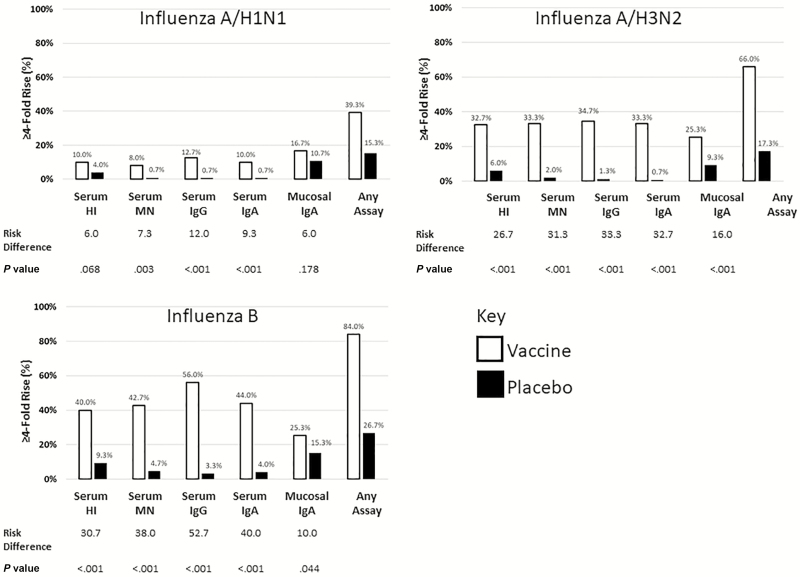
Post-vaccination 4-fold rises, by assays and influenza vaccine strains. All data points shown are the proportion (%) out of 150. Risk difference is defined as the difference between LAIV and the placebo. Abbreviations: HAI, hemagglutination inhibition assay; IgA, immunoglobulin A assay; IgG, immunoglobulin G assay; LAIV, live, attenuated influenza vaccine; MN, microneutralization assay.

Among all children at baseline, 192 (64%), 215 (72%), and 144 (48%) had pre-existing HAI titers ≥10 to the A/H1N1, A/H3N2, and B vaccine strains, respectively. LAIV and placebo groups were similar in baseline serostatus and prevaccination GMTs for each strain (Table 1). The primary immunogenicity outcomes of this study were serum HAI evaluations between vaccine groups (comparisons of GMT rise and 4-fold titer rise). The post-vaccination HAI GMT ratio of LAIV to placebo in participants with baseline seronegative status was 6.8 (95% CI 3.8–12.5) for A/H3N2, 2.7 (95% CI 1.9–3.9) for B, and 1.1 for A/H1N1 (95% CI 0.9–1.4). The post-vaccination GMT ratio of LAIV to placebo in participants with baseline seropositive status was 1.6 for A/H3N2 (95% CI 1.3-2), 1.6 for B (95% CI 1.2–2.1), and 1.4 for A/H1N1 (95% CI 1.1–1.8). Participants receiving LAIV had 4-fold rises in HAI titers for A/H1N1 (10.0%), A/H3N2 (32.7%), and B (40.0%), while participants receiving placebo had 4-fold rises in HAI titers for A/H1N1 (4.0%), A/H3N2 (6.0%), and B (9.3%; Figure 2). When we assessed 4-fold rises in titers for IgG, IgA, and MN between vaccine groups, we found LAIV was significantly more immunogenic for each vaccine virus (Figure 2).

**Table 1.** Serum and Mucosal Immune Responses in Children Aged 24 Through 59 Months Receiving Live, Attenuated Influenza Vaccine or Placebo

Assay/Influenza Subtype	Study Day	LAIV			Placebo			GMT Ratio, LAIV/Placebo	
		n	GMT	95% CI	n	GMT	95% CI	Ratio	95% CI
Serum HAI (Baseline Titer <10)									
A/H1N1	Pre	55	5.03	4.97–5.09	53	5.00	5.00–5.00	1.0	1.0–1.0
	Post	55	6.93	5.81–8.27	53	6.20	5.31–7.24	1.1	0.9–1.4
A/H3N2	Pre	40	5.02	4.98–5.07	45	5.00	5.00–5.00	1.0	1.0–1.0
	Post*	40	43.11	24.42–76.08	45	6.30	5.14–7.71	6.8	3.8–12.5
B	Pre	80	5.06	4.99–5.14	76	5.00	5.00–5.00	1.0	1.0–1.0
	Post*	80	18.18	13.19–25.04	76	6.63	5.61–7.85	2.7	1.9–3.9
Serum HAI (Baseline Titer ≥10)									
A/H1N1	Pre	95	37.32	31.84–43.74	97	36.32	31.35–42.08	1.0	0.8–1.3
	Post*	95	50.88	42.99–60.20	97	36.23	30.94–42.42	1.4	1.1–1.8
A/H3N2	Pre	110	44.66	38.80–51.40	105	49.25	42.92–56.51	0.9	0.7–1.1
	Post*	110	84.43	73.58–96.87	105	51.72	44.01–60.79	1.6	1.3–2.0
B	Pre	70	31.84	26.66–38.02	74	34.25	28.69–40.88	0.9	0.7–1.2
	Post*	70	63.37	50.89–78.91	74	40.24	32.54–49.76	1.6	1.2–2.1
Serum MN (all)									
A/H1N1 (California)	Pre	150	47.92	36.99–62.09	150	56.67	44.00–72.98	0.8	0.6–1.2
	Post	150	62.87	47.82–82.67	150	58.34	45.46–74.87	1.1	0.7–1.6
A/H3N2 (Perth)	Pre	150	119.41	91.51–155.81	150	135.58	106.03–173.36	0.9	0.6–1.3
	Post*	150	369.88	300.15–455.80	150	134.54	104.66–172.94	2.7	2.0–3.8
B (Brisbane)	Pre	150	15.44	12.60–18.93	150	15.66	12.77–19.21	1.0	0.7–1.3
	Post*	150	48.95	38.52–62.21	150	17.46	14.15–21.56	2.8	2.0–3.9
Serum IgA (all)									
A/H1N1 (California)	Pre	150	183.19	152.36–220.26	150	221.40	187.99–260.75	0.8	0.6–1.1
	Post	150	267.59	219.54–326.14	150	217.35	183.85–256.95	1.2	1.0–1.6
A/H3N2 (Perth)	Pre	150	144.73	124.18–168.67	150	143.40	122.97–167.22	1.0	0.8–1.3
	Post*	150	338.70	286.82–399.97	150	142.73	122.13–166.81	2.4	1.9–3.0
B (Brisbane)	Pre	150	129.53	112.16–149.60	150	136.92	117.62–159.39	0.9	0.8–1.2
	Post*	150	376.68	317.98–446.21	150	145.40	124.55–169.73	2.6	2.1–3.3
Serum IgG (all)									
A/H1N1 (California)	Pre	150	4145.12	3209.80–5353.00	150	5103.26	4075.14–6390.78	0.8	0.6–1.1
	Post	150	5916.51	4663.22–7506.65	150	5103.26	4070.59–6397.91	1.2	0.8–1.6
A/H3N2 (Perth)	Pre	150	3867.53	3056.54–4893.71	150	4986.68	4130.67–6020.08	0.8	0.6–1.0
	Post*	150	10640.00	9392.35–12 053.4	150	5079.70	4224.08–6108.64	2.1	1.7–2.6
B (Brisbane)	Pre	150	2120.99	1691.42–2659.66	150	2211.07	1768.15–2764.93	1.0	0.7–1.3
	Post*	150	9051.01	7931.98–10 327.9	150	2358.84	1868.50–2977.85	3.8	2.9–5.0
Mucosal IgA (all)									
A/H1N1 (California)	Pre	150	0.48	0.39–0.59	150	0.51	0.41–0.64	0.9	0.7–1.3
	Post*	150	0.61	0.48–0.76	150	0.43	0.34–0.55	1.4	1.0–1.9
A/H3N2 (Perth)	Pre	150	0.58	0.48–0.71	150	0.55	0.44–0.67	1.1	0.8–1.4
	Post*	150	1.14	0.91–1.41	150	0.51	0.41–0.63	2.2	1.6–3.0
B (Brisbane)	Pre	150	0.41	0.33–0.51	150	0.37	0.29–0.48	1.1	0.8–1.5
	Post*	150	0.82	0.64–1.05	150	0.40	0.31–0.52	2.0	1.4–2.9

Pre indicates a day 0 blood draw; post indicates a day 21 blood draw.

*= P <.05 for T Test log10 Titer, LAIV vs Placebo.

Abbreviations: CI, confidence interval; GMT, geometric mean titers; HAI, hemagglutination inhibition assay; IgA, immunoglobulin A assay; IgG, immunoglobulin G assay; LAIV, live, attenuated influenza vaccine; MN, microneutralization assay.

The post-vaccination GMT ratio of LAIV to placebo by immunoassay and vaccine strains are in Table 1. There were significantly higher day 21 GMTs for the LAIV group, as compared to the placebo group, in all serum and mucosal immunoassays for A/H3N2 and B, as measured by the *t* test of log_10_ titers (for each comparison, *P* < .0001, GMT ratio >2.0). Among immunoassays for A/H1N1, only the mucosal IgA GMT was significantly higher than placebo at day 21 (*P* = .0465).

The percentage of participants in each study arm achieving 4-fold rises in titers for each immunoassay or for any immunoassay is presented in Figure 2.

### Viral Shedding Results

Prior to vaccination, no participants had evidence of the LAIV virus from NPW specimens. Within the week following vaccination, 117 (78.0%) of LAIV recipients had evidence of at least 1 detectable vaccine virus, including 68 (45.3%) for A/H3N2 and 101 (67.3%) for influenza B; however, no participants (0.0%) had evidence of influenza A/H1N1 (Table 2). There were 7 additional LAIV recipients who had evidence of vaccine influenza A that was unsubtypable or inconclusive. There was 1 placebo recipient who had evidence at day 2 of vaccine-type influenza B, which did not persist to days 4 or 7. There were no other LAIV virus detections in placebo recipients during the study. Wild-type influenza B/Victoria virus was detected in 1 LAIV recipient, on day 7. Wild-type influenza B/Yamagata viruses were also detected in 3 participants in the placebo group, at days 0 (1), 2 (1), 4 (1), and 7 (3).

**Table 2.** Vaccine Virus Detections in Live, Attenuated Influenza Vaccine Recipients Aged 24 Through 59 Months

Bangladesh^a^2012–2013	LAIV Group Vaccine VirusDay 2	LAIV Group Vaccine VirusDay 4	LAIV Group Vaccine VirusDay 7	LAIV Group Vaccine Virus Any Day
Any vaccine strain	108/150 (72.0%)	97/150 (64.7%)	72/150 (48.0%)	117/150 (78.0%)
Vaccine A/H1N1	0/150 (0.0%)	0/150 (0.0%)	0/150 (0.0%)	0/150 (0.0%)
Vaccine A/H3N2	60/150 (40.0%)	50/150 (33.3%)	23/150 (15.3%)	68/150 (45.3%)
Vaccine B	89/150 (59.3%)	73/150 (48.7%)	59/150 (39.3%)	101/150 (67.3%)

Abbreviation: LAIV, live, attenuated influenza vaccine.

^a^The vaccine used was the World Health Organization–recommended formulations for the Northern Hemisphere 2012–2013 season (A/California/7/2009 [H1N1] pdm09-like virus; A/Victoria/361/2011 [H3N2]-like virus; and B/Wisconsin/1/2010-like virus).

### Correlation Between Outcome Measures

There was moderate to good agreement (Kappa ≤0.73) between strain-specific serum immunoassay antibody responses (Table 3), particularly for A/H3N2. There was little agreement (Kappa ≤0.23) between strain-specific mucosal and serum antibody responses, and little agreement (Kappa ≤0.33) between the strain-specific viral shedding and antibody responses.

**Table 3.**
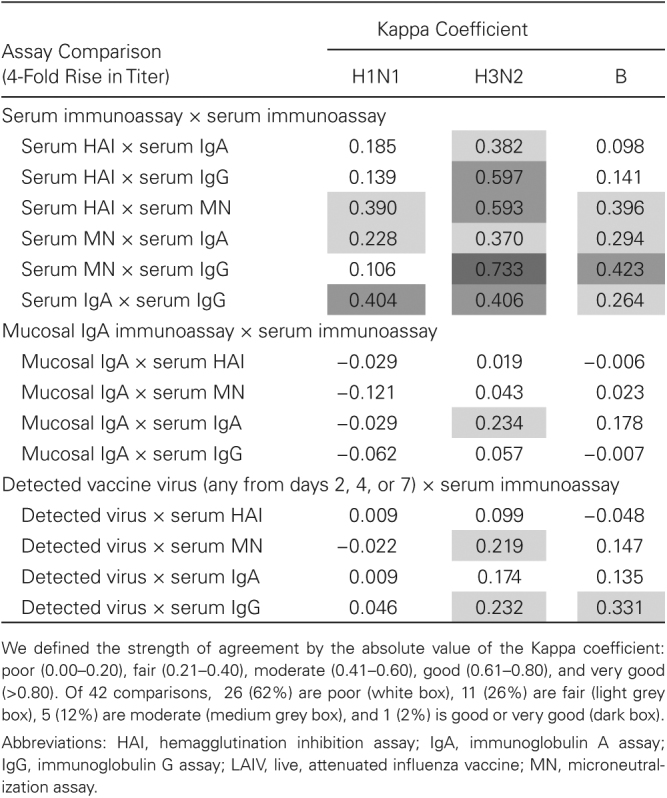
Correlations Among Post-Vaccination Immunoassays Achieving 4-Fold Rise in Titer and Any Vaccine Virus Detections in LAIV Recipients Aged 24 Through 59 Months

## DISCUSSION

In this Phase II RCT of 2012–2013 LAIV among young children in Bangladesh, immunologic assays yielded measureable results for the A/H3N2 and B components, but the response was uniformly low for the A/H1N1 component. Likewise, there was evidence for the infectivity of the A/H3N2 and B strains, but not for the A/H1N1 vaccine virus. While the protective mechanisms induced by vaccination with LAIV are incompletely understood, the very limited measurable immunological responses in the assays against the A/H1N1 vaccine virus in this trial are concerning, particularly given the recent challenges identified with the Ann Arbor–backbone LAIV A/H1N1 component [7, 9, 11, 14, 22, 23].

While LAIVs have no correlate of protection, higher HAI GMTs in children generally confer more clinical protection from influenza [24]. We detected significant HAI antibody responses among children who were seronegative and seropositive at baseline, except for the H1N1-unexposed group. The post-vaccination HAI GMTs among seropositive children were 2–7 times greater than among seronegative children. While a single dose of Ann Arbor or Russian LAIV has been shown to provide clinical protection against laboratory-confirmed influenza [12, 25, 26], it is possible that a series of 2 LAIV doses for vaccine-naive individuals would have been more efficacious. Increasing the primary vaccine series from 1 dose to 2 would introduce additional feasibility challenges to implementation in LMICs, however.

In a subsequent Phase III clinical-efficacy trial in the same Bangladesh community the following year (2013–2014), we demonstrated efficacy against laboratory-confirmed clinical influenza, with vaccine efficacy against all circulating strains of 41.0% (95% CI 28.0–51.6) and vaccine efficacy against vaccine-matched strains of 57.5% (95% CI 43.6–68.0; Table 4) [12]. In that study, we demonstrated a LAIV efficacy specifically against A/H1N1 of 50.0% (95% CI 9.2–72.5). The A/H1N1 component and the manufacturing processes had not changed between the 2 study years, further emphasizing a lack of correlation between standard immunologic measurements and vaccine efficacy for LAIV [27].

**Table 4.** Comparison of Current Study Data to Previously Published, Randomized, Clinical Trials of Russian-Backbone Live, Attenuated Influenza Vaccine in Bangladesh and Senegal

	Efficacy Evaluation			Shedding Evaluation			
Trial	LAIV Cases	Placebo Cases	Vaccine Efficacy (95% CI)	LAIV Group Vaccine VirusDay 2	LAIV Group Vaccine VirusDay 4	LAIV Group Vaccine VirusDay 7	LAIV Group Vaccine Virus Any Day
Bangladesh^a^ 2012–2013							
Any vaccine strain	...	...	...	108/150 (72.0%)	97/150 (64.7%)	72/150 (48.0%)	117/150 (78.0%)
Vaccine A/H1N1	...	...	...	0/150 (0.0%)	0/150 (0.0%)	0/150 (0.0%)	0/150 (0.0%)
Vaccine A/H3N2	...	...	...	60/150 (40.0%)	50/150 (33.3%)	23/150 (15.3%)	68/150 (45.3%)
Vaccine B	...	...	...	89/150 (59.3%)	73/150 (48.7%)	59/150 (39.3%)	101/150 (67.3%)
Bangladesh^b,c^ 2013–2014 [12]							
All strains	170 (15%)	144 (25%)	41.0% (28.0–51.6)	...	...	...	...
All vaccine strains	79 (7%)	93 (16%)	57.5% (43.6–68.0)	...	...	...	...
Vaccine A/H1N1	21 (2%)	21 (4%)	50.0% (9.2–72.5)	...	...	...	...
Vaccine A/H3N2	57 (5%)	72 (12%)	60.4% (44.8–71.6)	...	...	...	...
Vaccine B	2 (0%)	1 (0%)	0.0% (−1001.0 to 90.0)	...	...	...	...
Mismatched B	58 (5%)	31 (5%)	6.5% (−43.0 to 38.8)	...	...	...	...
Senegal^b,c^ 2013–2014 [13]							
All strains	210 (18%)	105 (18%)	0.0% (−26.4 to 20.9)	...	...	...	...
All vaccine strains	100 (9%)	47 (8%)	−6.1 (−50.0 to 25.0)	48/65 (74%)	39/66 (59%)	...	55/66 (83%)
Vaccine A/H1N1	79 (7%)	36 (6%)	–9.7% (−62.6 to 26.1)	12/65 (19%)	3/66 (5%)	...	14/65 (22%)
Vaccine A/H3N2	3 (<1%)	0 (0%)	...	31/65 (48%)	18/66 (27%)	...	34/65 (52%)
Vaccine B	20 (2%)	11 (2%)	9.5% (−88.9 to 56.6)	34/65 (52%)	28/65 (43%	...	42/64 (66%)
Mismatched B	115 (10%)	62 (11%)	7.3% (−26.3 to 31.9)	...	...	...	...

Significant figures are different because of differences in published results among cited studies. Studies included are all the published randomized clinical trials of Russian-backbone LAIV with laboratory-confirmed endpoints.

Abbreviations: CI, confidence interval; LAIV, live, attenuated influenza vaccine.

^a^The vaccine used was the World Health Organization–recommended formulation for the Northern Hemisphere 2012–2013 season (A/California/7/2009 [H1N1] pdm09-like virus; A/Victoria/361/2011 [H3N2]-like virus; and B/Wisconsin/1/2010-like virus).

^b^The vaccine used was the World Health Organization–recommended formulation for the Northern Hemisphere 2013–2014 season (A/California/7/2009 [H1N1] pdm09-like virus; A/Victoria/361/2011 [H3N2]; and a B/Massachusetts/2/2012-like virus.)

^c^

Efficacy results are from the per protocol populations.

The only other published clinical-efficacy RCT of SIIL LAIV was from Senegal (Table 4) [12]. This Phase III trial was conducted in 2013–2014, with a similar design to the Bangladesh Phase III trial. The same lot of lyophilized vaccines was used for both studies. Unlike the Bangladesh Phase III trial, the Senegal trial had a small subset of participants (68 LAIV and 32 placebo) enrolled in additional safety and shedding evaluations for the week post-vaccination. This trial failed to demonstrate a significant vaccine efficacy. As most confirmed influenza illnesses were either A/H1N1 or a mismatched B-lineage virus, it was not possible to determine whether the lack of efficacy was a strain-specific effect or a broader vaccine effect in Senegal. The shedding component of the Senegal study demonstrated that all 3 vaccine strains were present in nasal swab specimens post-vaccination. Of LAIV recipients, 83% had evidence of at least 1 vaccine virus post-vaccination, including 52% with A/H3N2, 66% with B, and 22% with A/H1N1. The circulating A/H1N1 virus was well matched to the vaccine. The researchers were unable to identify a specific cause for the observed lack of efficacy.

Since 2014, observational influenza vaccine studies have identified decreased Ann Arbor–backbone LAIV effectiveness against A/H1N1. In 2016, the manufacturers of the Ann Arbor LAIV reported that the vaccine effectiveness of their product against A/H1N1 had decreased with increased storage temperatures [28], and mutation in the WHO-recommended A/H1N1 vaccine component could decrease the thermal stability of the strain [29]. However, replacement of the attenuated strain with a more thermal-stable virus did not improve vaccine effectiveness. In 2015–2016, the CDC Influenza Vaccine Effectiveness Network measured no significant effectiveness of LAIV in children, while inactivated influenza vaccines performed according to expectations [7]. This study and others done at the same time in North America and Europe implicated the A/H1N1 component of the vaccine [6, 7]. AstraZeneca/MedImmune has described data relevant to several hypotheses that may explain the decreased vaccine effectiveness of LAIV, including reduced replicative fitness of the A/H1N1 component strains, vaccine virus interference, and differences in prior influenza vaccine receipt [14].

For the 2017–2018 season, AstraZeneca/MedImmune changed the A/H1N1 strain of its product from A/Bolivia to A/Slovenia. In July 2018, Public Health England reported LAIV vaccine effectiveness against A/H1N1 in children was 90.3% (95% CI 16.4–98.9%) [30].

While the Ann Arbor–backbone and Russian-backbone LAIVs are different products, they have some similarities. Both based the A/H1N1 vaccine component on the same wild-type virus during the time of the Bangladesh and Senegal studies, suggesting SIIL LAIV could have had similar thermal-stability issues. However, the cold chain, vaccine reconstitution, and administration were carefully monitored during the Bangladesh and Senegal trials, and there were no significant temperature excursions [12, 13]. The potency of shelf-stocks of the vaccine had been assessed by the manufacturer, which found no cause for the discrepant 2013–2014 trial result. While some Senegalese children had received an influenza vaccine before and none of the Bangladeshi participants had, specific subset analyses in Senegal categorizing participants by prior vaccine exposure did not show any differences in vaccine effectiveness [13]. As with the Ann Arbor LAIV, it is possible that decreased replicative fitness or vaccine-virus interference were playing a role in the decreased vaccine performance of the A/H1N1 component of the SIIL LAIV. SIIL has changed the influenza A/H1N1 seed virus, and the manufacturer expects to have better replicative fitness and antigenicity in a trivalent/quadrivalent vaccine preparation (personal communication, Leena Yeolekar, SIIL, [8 February 2018]). It has also embarked on a series of animal studies to explore the hypotheses that either vaccine interference or decreased fitness may have contributed to our findings (personal communication, Leena Yeolekar, SIIL). A large, clinical-efficacy RCT of SIIL LAIV vs an inactivated influenza vaccine is wrapping up in India and may provide further insight soon (CTRI/2015/06/005902).

Our study should be interpreted in the context of its limitations. We studied the effect of 1 dose of Russian-backbone LAIV, as per product instructions [16]. A second dose, as is recommended in some countries for all vaccines for vaccine-naive children <9 years, may have improved vaccine performance. There were high rates of 2009 A/H1N1 infection documented in Bangladeshi children after the 2009 pandemic, suggesting the potential for preexisting immunity [31]; however, only 36% of participants had serological evidence of prior 2009 A/H1N1 exposure, lower than serological evidence of exposure to the other vaccine components. None of the immunological or shedding evaluations we used are correlated with clinical protection. The absence of a detectable immune response or vaccine virus post-vaccination does not necessarily equate with the absence of priming or a vaccine effect. Finally, we did not conduct potency testing of unused vaccine doses from the trial.

The WHO has determined that there is an unmet global health need for influenza vaccines that prevent severe illness in young children and that are programmatically suitable for use in low-resource settings [2]. LAIVs have the potential to address this unmet need, but urgent efforts are required to understand and to correct possible problems seen with A/H1N1 components in both vaccines in use today.

## Notes

***Acknowledgements.*** The authors thank Kristin Bedell, Jodi Feser, the GVKBio Trial Monitors, and Emmes for their statistical services. They thank F. Liani Gross, Zhunan Li, Stacie Jefferson, Jessica Trost, and David Wang from the Influenza Division of the Centers for Disease Control and Prevention for their assistance in conducting serum immunogenicity assays (hemagglutination inhibition, microneutralization, immunoglobulin G, and immunoglobulin A enzyme-linked immunosorbent assays). They also thank the members of the study’s international data safety monitoring board and local data safety monitoring board. Finally, they thank all the study participants and their families; the staff at the Kamalapur Field Site for their dedication to their patients; and study staff at the laboratories and administrative offices of International Centre for Diarrheal Disease Research, Bangladesh, Dhaka.

***Disclaimer.*** The findings and conclusions in this report are those of the authors. The authors alone are responsible for the views expressed in this publication and they do not necessarily represent the decisions or policies of their organizations. The funder and vaccine manufacturer had no role in the design and conduct of the study; collection, management, analysis, and interpretation of the data; or preparation, review, or approval of the manuscript.

***Financial support.*** This work was supported by funding from the Bill & Melinda Gates Foundation, which provides financial support to PATH’s Influenza Vaccine Project (grant number OPP48805). Serum Institute of India, Ltd., donated the study vaccine and placebo.

***Potential conflicts of interest.*** J. R. O., K. M. N., M. Z. R., and M. R. report a grant from the Centers for Disease Control and Prevention. J. M. K. has 2 patents issued: “Preparation and use of recombinant influenza A virus M2 construct vaccine” and “An effective vaccine against pandemic strains of influenza viruses.” All other authors: No reported conflicts. All authors have submitted the ICMJE Form for Disclosure of Potential Conflicts of Interest. Conflicts that the editors consider relevant to the content of the manuscript have been disclosed.
